# Novel Three-Finger Neurotoxins from *Naja melanoleuca* Cobra Venom Interact with GABA_A_ and Nicotinic Acetylcholine Receptors

**DOI:** 10.3390/toxins13020164

**Published:** 2021-02-20

**Authors:** Lina Son, Elena Kryukova, Rustam Ziganshin, Tatyana Andreeva, Denis Kudryavtsev, Igor Kasheverov, Victor Tsetlin, Yuri Utkin

**Affiliations:** 1 Shemyakin-Ovchinnikov Institute of Bioorganic Chemistry, ul. Miklukho-Maklaya 16/10, 117997 Moscow, Russia; lina.son@phystech.edu (L.S.); evkr@mail.ru (E.K.); rustam.ziganshin@gmail.com (R.Z.); damla-sofia@yandex.ru (T.A.); kudryavtsevden@gmail.com (D.K.); shak_ever@yahoo.com (I.K.); victortsetlin3f@gmail.com (V.T.); 2 Moscow Institute of Physics and Technology, 141700 Dolgoprudny, Russia; 3 Institute of Molecular Medicine, Sechenov First Moscow State Medical University, ul. Trubetskaya 8, bld. 2, 119991 Moscow, Russia

**Keywords:** binding sites, cobra venom, GABA_A_ receptor, neurotoxin, three-finger toxin, nicotinic acetylcholine receptor, acetylcholine binding protein

## Abstract

Cobra venoms contain three-finger toxins (TFT) including α-neurotoxins efficiently binding nicotinic acetylcholine receptors (nAChRs). As shown recently, several TFTs block GABA_A_ receptors (GABA_A_Rs) with different efficacy, an important role of the TFTs central loop in binding to these receptors being demonstrated. We supposed that the positive charge (Arg36) in this loop of α-cobratoxin may explain its high affinity to GABA_A_R and here studied α-neurotoxins from African cobra *N. melanoleuca* venom for their ability to interact with GABAARs and nAChRs. Three α-neurotoxins, close homologues of the known *N. melanoleuca* long neurotoxins 1 and 2, were isolated and sequenced. Their analysis on *Torpedo* *californica* and α7 nAChRs, as well as on acetylcholine binding proteins and on several subtypes of GABA_A_Rs, showed that all toxins interacted with the GABA_A_R much weaker than with the nAChR: one neurotoxin was almost as active as α-cobratoxin, while others manifested lower activity. The earlier hypothesis about the essential role of Arg36 as the determinant of high affinity to GABA_A_R was not confirmed, but the results obtained suggest that the toxin loop III may contribute to the efficient interaction of some long-chain neurotoxins with GABA_A_R. One of isolated toxins manifested different affinity to two binding sites on *Torpedo* nAChR.

## 1. Introduction

Ligand-gated ion channels are classified into several families of membrane-bound receptors. The Cys-loop family of pentameric receptors is represented widely in the muscle and nervous systems as well as in immune and other cells and plays prominent roles. The Cys-loop family of vertebrates includes nicotinic acetylcholine receptor (nAChR), as well as serotonin type 3, γ-aminobutyric acid (GABA_A_R), and glycine receptors. The receptors of this family are characterized by a conserved sequence of 13 amino acid residues confined by two cysteines forming a disulfide bond (Cys loop) in the *N*-terminal extracellular domain of each subunit [1]. Among these receptors, excitatory nAChRs are the targets of numerous natural compounds including toxins from snake venom [2,3]. Snake venom neurotoxins of the three-finger toxin (TFT) family efficiently inhibit some nAChR subtypes. For example, α-bungarotoxin (α-Bgt) from krait *Bungarus multicinctus* venom binds muscle-type as well as neuronal α7 and α9 nAChRs with nanomolar affinities and blocks ion current through the ion channel of these receptors. α-Cobratoxin (α-Ctx) from cobra *Naja kaouthia* venom exhibits similar effects. Based on location and function, nAChRs can be divided into muscle and neuronal ones [4,5,6]. Neuronal nAChRs are localized in the central and peripheral nervous system and are involved in the transmission of fast nerve impulses. Muscle-type nAChRs are located on the postsynaptic membranes of the neuromuscular junction and transmit signals for muscle contraction. Each muscle-type nAChR has two copies of α1 and one each of the β1, δ, and γ subunits (in the embryonic receptor) or ε subunit (in the adult form). In this receptor type, two binding sites for agonists and competitive antagonists are located in the extracellular domain at the interfaces of the α1–δ and α1–γ (or α1–ε) subunits [5]. Two binding sites have different affinity to acetylcholine, the α–γ site possessing a ∼35–40-fold higher affinity for acetylcholine than the α–δ and α–ε sites [7]. Some natural toxins, including several α-conotoxins, waglerins, and few TFTs, bind with different affinities to these two binding sites as well. Thus, a short α-neurotoxin from cobra *N. mossambica mossambica* (NmmI) was shown to bind with high affinity to α–γ and α–δ subunit interfaces but had a markedly reduced affinity to the α–ε interface [8]. Furthermore, the mutation of Lys27 to Glu27 in NmmI affected binding at the α–γ site more than the α–δ site [9] making this mutant selective for α–δ site. The similar results were obtained for two mutants of α-Ctx K23E and K49E, which demonstrated the different affinities to two toxin-binding sites on *Torpedo* nAChR with higher and lower affinities at the α–δ and α–γ sites, respectively [10]. The data for nonconventional TFT, candoxin from *B. candidus* also suggest its differential affinity for the α–γ or α–δ sites at the muscle nAChR [11]. We have found recently that so called αδ-bungarotoxins from *B. candidus* venom show different affinity to two binding sites in muscle-type nAChRs, manifesting higher activity at the interface of α–δ subunits [12].

Until recently α-Bgt and α-Ctx were considered as very specific markers of nAChRs. However, the works of our and other groups [13,14,15] have shown that these toxins inhibit also GABA_A_R. Similarly to nAChRs, each GABA_A_R is composed of five subunits, including most frequently two α subunits, two β subunits, and one from *γ*, *δ*, *ε*, *θ*, or *π* subunit. However, contrary to nAChRs which are cation channels, GABA_A_R are chloride channels. In mammals, 19 different subunits (six α, three β, three γ, δ, ε, θ, π, and three ρ) are known; they form a wide variety of GABA_A_ receptor subtypes with distinct subunit composition and unique pharmacological properties [16]. In the brain, the most abundant GABA_A_R isoforms are αβγ and αβδ [17]. GABA_A_ αβγ receptors are widely distributed in the brain, while αβδ receptors constitute only a small proportion [18]. Among α subunits, the most abundant is α1 which is often colocalized with highly expressed β2 and γ2 subunits [18]. The α2 and α3 subunits are less abundant. Among the β subunits, β2 is most abundant, β3 is reasonably highly expressed, and β1 is least common. Up to 80% of GABA_A_Rs contain the γ2 subunit [16]. In the brain, α1β2γ2 is the most common isoform. At present, several clinically used compounds target GABA_A_R and still there is a need in finding new molecules for potential design of novel drugs.

Acetylcholine binding proteins (AChBPs), soluble proteins mostly from mollusks, are remarkable structural homologues of the ligand-binding domains of all Cys-loop receptors [19]. While transmembrane and intracellular motifs are absent in these proteins, the important elements necessary for ligand binding including the C- and F-loops are structurally conserved. This fact made AChBPs and their mutants perfect tools for structural studies on pentameric ligand-gated ion channels. The fact that many studies have revealed some inconsistency in the activity profiles on nAChR and AChBP for a number of ligands was compensated by high-resolution structures of complexes of these ligands with AChBPs. Based on these data, more reliable models of the complexes of the corresponding ligands with the full-length nAChRs were built in order to identify the key amino acid residues and molecular mechanisms that determine the receptor-ligand interactions. In particular, the molecular basis for the high selectivity of α-conotoxin LvIA for α3β2 nAChR [20] or the difference in affinity of two other α-conotoxins’ analogues to human and rat α7 nAChR [21] were explained. At present, AChBPs from the mollusks *Aplysia californica* and *Lymnaea stagnalis* [22] are widely used, the former is closer pharmacologically to homooligimeric nAChRs while the latter is closer to heterooligomeric receptors.

α-Bgt, being a very efficient blocker of nAChRs, showed fairly weak activity on GABA_A_R inhibiting α1β3γ2 receptors by only 19% at 10 µM [15]. In contrast, α-Ctx inhibited GABA_A_R quite effectively manifesting half-maximal inhibitory concentration (IC_50_) of 236 nM at α1β3γ receptor [15]. Some other snake neurotoxins inhibited GABA_A_R as well, although not so effectively as α-Ctx. It was suggested that Arg36, present in α-Ctx and being valine in this position of α-Bgt, might be responsible for efficient interaction of α-Ctx with GABA_A_R [6]. In order to test this hypothesis, α-Ctx analogues with or without Arg36 need to be used. Although the most straightforward way would be testing the α-Ctx analogue(s) obtained through site-directed mutagenesis at position occupied by Arg36, there are some problems that should be solved on this way. First one is that α-Ctx contains five disulfide bridges the correct formation of which in mutant should be proved. This may require either determination of spatial structure (e.g., by X-ray or NMR) or combined used of selective disulfide modification and peptide mass fingerprinting. Moreover, the cleavage of some chimeric proteins often used to obtain the protein of interest or direct expression of the protein may add extra amino acid residues at *N*-terminus, which may influence the biological activity. We believed that use of natural α-Ctx analogues containing either Arg36 or other residue at this position may be the easiest way to test our hypothesis. If natural α-Ctx analogue with Arg36 is not as active as α-Ctx itself, then this arginine residue is not the only determinant of high α-Ctx affinity to GABA_A_R. In this respect, our attention was attracted to the cobra *N. melanoleuca* venom in which the presence of two highly homologous long chain neurotoxins 1 and 2 was shown [23,24]: neurotoxin 1 contained valine and neurotoxin 2 contained arginine residue at the position corresponding to Arg36 in α-Ctx. They seemed a good pair to clarify the role the respective residue.

In the present work from *N. melanoleuca* venom we isolated three α-neurotoxins (two with arginine and one with valine at the position corresponding to Arg36 in α-Ctx) and one muscarinic toxin-like protein. Only one neurotoxin (designated here as TX-NM4) was identical to the earlier described *N. melanoleuca* neurotoxin 2, another (TX-NM3-1) was its analogue with 5 substitutions, while instead of neurotoxin 1 we isolated its analogue with 3 substitutions. Analyzing binding of the isolated toxins to GABA_A_Rs and inhibition of the ion currents elicited by GABA, we did not find any evidence for the role of Arg36. However, molecular modeling showed that α-Ctx loop III had contact with α1 subunit of GABA_A_R and this contact might contribute to the efficient binding of the toxin to the receptor. Thus, our work expands the range of TFTs capable of inhibiting the GABA_A_R subtypes. In addition, we found that one *N. melanoleuca* toxin (Tx-NM2) showed unequal affinities for the two ligand-binding sites in the *Torpedo californica* nAChR and thus widened the range of naturally occurring TFTs that differently interact with the two binding sites in muscle-type nAChRs. Keeping in mind the future modeling and structural studies of the interaction of *N. melanoleuca* toxins with nAChRs and GABA_A_Rs, we investigated the interaction of these toxins with AChBPs from *A. californica* and *L. stagnalis* as plausible models. Tx-NM2 was found to be the most potent in binding with both AChPBs.

## 2. Results

### 2.1. Isolation and Characterization of Naja melanoleuca Toxins

**Figure 1:**
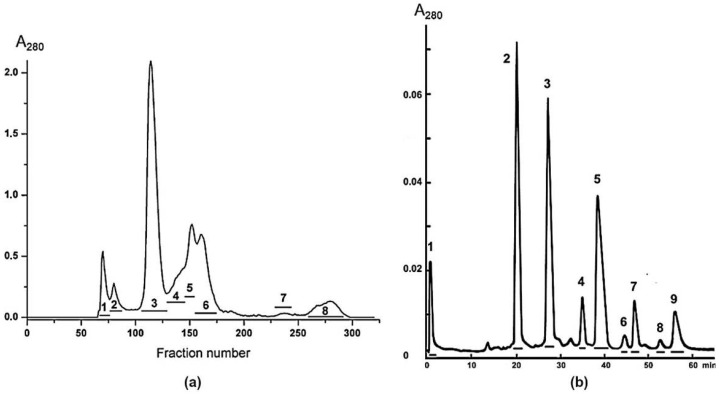
Isolation of *N. melanoleuca* neurotoxins. (**a**) Separation of the crude *N. melanoleuca* venom by gel-filtration on Sephadex G50 column. (**b**) Separation of fraction 6 by ion exchange chromatography on HEMA BIO 1000CM column.

To isolate toxins, a three-step chromatographic procedure was used. Gel filtration on Sephadex G50 was the first step (Figure 1a). The analysis of obtained fractions by mass spectrometry showed that fraction 6 contained proteins with molecular masses in the range of 6–8 kDa. In cobra venom, these masses are characteristic of three-finger toxins. As mentioned in introduction, we were interested in long neurotoxins 1 and 2 possessing the molecular masses of 8040.76 and 7756.59 Da, respectively. Therefore, the fraction 6 containing three-finger toxins was further separated by ion exchange chromatography on HEMA BIO 1000CM column (Figure 1b) and fractions obtained were analyzed by mass spectrometry. Fractions 2, 3, and 4 containing toxins with molecular masses of about 8 kDa were further purified by reversed phase HPLC (Figure S1). As a result, one toxin was obtained from fraction 2 (Tx-NM2), two toxins from fraction 3 (Tx-NM3-1, Tx-NM3-2) and one toxin from fraction 4 (Tx-NM4). Finally, four toxins with molecular masses of 8030.68 (Tx-NM2), 7787.577 (Tx-NM3-1), 7441.497 (Tx-NM3-2), and 7756.581 (Tx-NM4) Da were purified (Table 1, Figure S2).

**Figure 2:**
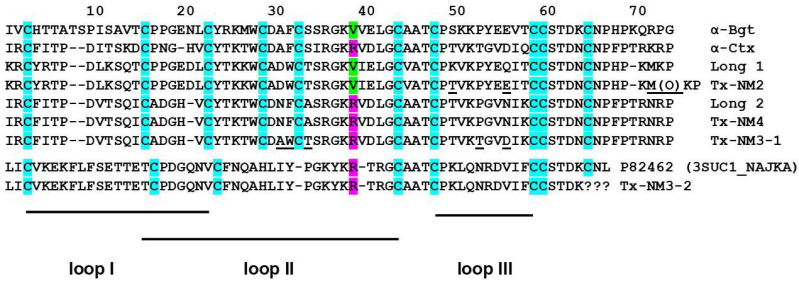
Alignment of amino acid sequences. Long 1 is long type α-neurotoxin 1 from *N. melanoleuca* P01383 (3L21_NAJME), Long 2 is long type α-neurotoxin 2 from *N. melanoleuca* P01388 (3L22_NAJME), toxin P82462 (3SUC1_NAJKA) is muscarinic toxin like protein from *N. kaouthia* venom. All toxins have three loops stabilized by five disulfide bonds. The residues different in α-neurotoxin 1 and Tx-NM2 as well as α-neurotoxin 2 and Tx-NM3-1 are underlined. Arginine and valine residues corresponding to position 36 in α-Ctx sequence are shown in lilac and green, respectively.

The amino acid sequences of isolated toxins were determined by tandem mass spectrometry. For this purpose, the toxins were reduced, carbamidomethylated and digested with trypsin or chymotrypsin. The peptides obtained were analyzed by LC-MS with simultaneous de novo mass spectrometry sequencing. As a result, the complete amino acid sequences of Tx-NM2, Tx-NM3-1, and Tx-NM4 as well as partial amino acid sequence of Tx-NM3-2 were determined (Figure 2).

It was found that the amino acid sequence of Tx-NM4 exactly corresponds to that of long type α-neurotoxin 2 from *N. melanoleuca* (Uniprot accession number P01388). The sequence of Tx-NM2 differs from that of *N. melanoleuca* long type α-neurotoxin 1 (P01383) in three positions: at position 50, Lys residue is changed to Thr, Gln56 to Glu, and Met72 is oxidized. Tx-NM3-1 is very similar to long type α-neurotoxin 2 (P01388), but its sequences differ in 5 positions (Figure 2). We were not able to determine the amino acid sequence of Tx-NM3-2 at the short *C*-terminal fragment, however this toxin is very similar to muscarinic toxin-like protein 1 (P82462) from *N. kaouthia* cobra venom (Figure 2).

### 2.2. Biological Activity of N. melanoleuca Toxins

#### 2.2.1. Interaction with GABA_A_R

The biological activity of isolated toxins was studied on rat GABA_A_Rs obtained by heterologous expression of different combination of α, β and γ subunits. The most common combinations of GABA_A_R subunits were used. They included α1β2γ2, α1β3γ2, and α3β2γ2 receptors. As concerns the inclusion of γ2 subunit, it has been shown earlier that increase in the ratio of mRNA/cDNA relative to α1 and β2 subunits (up to 10:1:1) resulted in more homogenous α1β2γ2 receptors in oocytes [25]. In our work, we used exactly this high γ2 subunit ratio relative α1 and β2 (10:1:1). Moreover, the presence of γ subunit in the receptors was proved in electrophysiology experiments as shown below.

- Competition of *N. melanoleuca* toxins with fluorescently labeled α-Ctx

**Figure 3:**
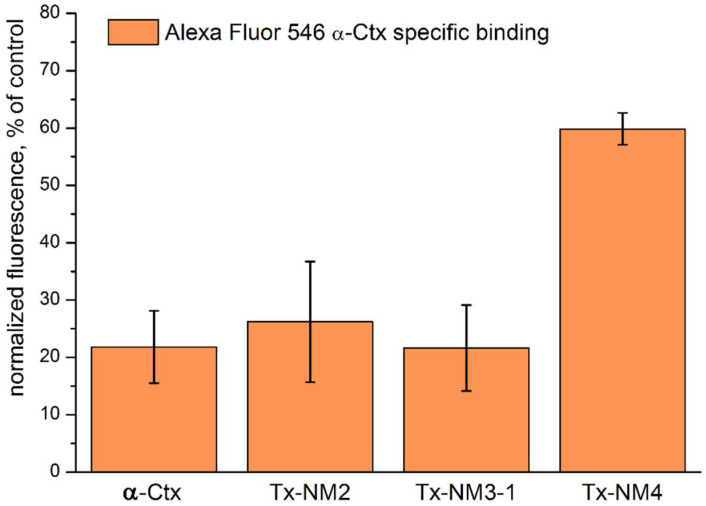
Inhibition of Alexa Fluor 546 α-Ctx staining of Neuro 2a cells expressing α1β3γ2 GABA_A_R by *N. melanoleuca* toxins at concentrations of 10 μM. Data are shown as percent of control staining without toxins and presented as Mean ± SEM (*n* = 3).

In this work, we used the most prevalent types of GABA_A_Rs. We have shown previously that α-Ctx labelled with Alexa Fluor 546 fluorophore efficiently stained Neuro 2a cells heterologously expressing α1β3γ2 GABA_A_R and this staining was inhibited by native α-Ctx and some other toxins [15]. α-Ctx was shown to be the most active on this receptor type [15], and we used it first to test the activity of *N. melanoleuca* toxins (Figure 3). The activity of *N. melanoleuca* toxins on the α1β3γ2 receptor was tested with the mentioned fluorescence assay. It was found that at 10 μM Tx-NM2 and Tx-NM3-1 inhibited Alexa Fluor 546 α-Ctx binding to GABA_A_R as efficiently as native α-Ctx, while Tx-NM4 was less active (Figure 3).

- Electrophysiology measurements

**Figure 4:**
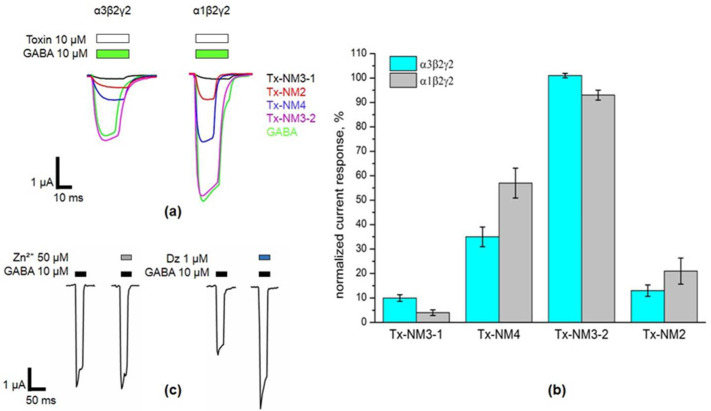
Activity testing of *N. melanoleuca* toxins by two-electrode voltage clamp (TEVC) on *Xenopus* oocytes expressing GABA_A_ α1β2γ2 and α3β2γ2 receptor subtypes. (**a**) Representative current traces illustrating inhibition of 10 μM GABA-evoked currents by *N. melanoleuca* toxins at 10 μM concentration. (**b**) Comparison of the activity of *N. melanoleuca* toxins at GABA_A_R. The response to GABA without toxins is taken as 100%. Data are shown for α1β2γ2 and α3β2γ2 GABA_A_R and presented as Mean ± SEM (*n* = 3). For toxins Tx-NM3-1, Tx-NM3-2, and Tx-NM4, the difference in interaction with two receptor subtypes is statistically significant. *p* < 0.05 according to Student’s *t* test. (**c**) Example current traces at α1β2γ2 GABA_A_R illustrating gamma subunit incorporation. GABA potency was not affected by 50 μM of Zn^2^⁺ but was enhanced by 1 μM of Diazepam (Dz).

**Figure 5:**
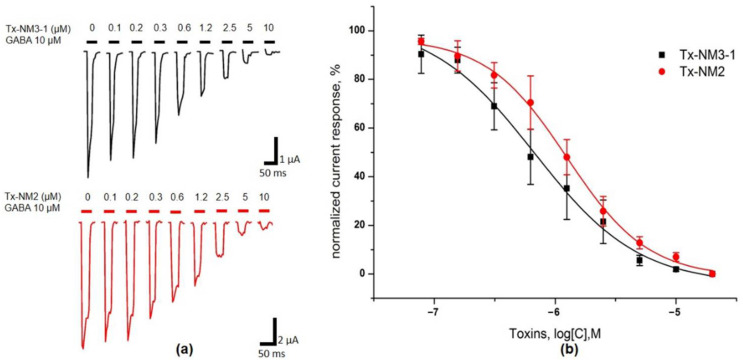
Activity testing of Tx-NM3-1 and Tx-NM2 at α1β3γ2 GABA_A_R expressed in *Xenopus* oocytes by TEVC. (**a**) Representative current traces elicited by 10 μM GABA in presence of Tx-NM3-1 and Tx-NM2 in 0–10 μM concentration range. (**b**) Dose–response curves showing the dependence of current elicited by 10 μM GABA on the concentration of toxins Tx-NM3-1 (squares) and Tx-NM2 (circles). The data are presented as Mean ± SEM (*n* = 3–5).

Functional activities of the isolated toxins were studied by two-electrode voltage clamp on mouse GABA_A_Rs heterlogously expressed in *Xenopus laevis* oocytes (Figure 4 and Figure 5). Three combinations of receptor subunits were used in this study: α1β2γ2, α3β2γ2, and α1β3γ2. The incorporation of γ subunit was confirmed in separate experiments which are shown for the α1β3γ2 receptor (Figure 4c). GABA-induced currents at the α1β3γ2 receptor were not inhibited by Zn^2+^ ions, while inhibition was observed at α1β3 receptors (not shown). The incorporation of γ subunit in α1β3γ2 receptors was further confirmed by their sensitivity to diazepam (Figure 4c).

First, we checked the activity of toxins on the common receptor types α1β2γ2 and α3β2γ2, α1β2γ2 being the most common isoform (Figure 4). Finally, the quantitative characteristics for binding (IC_50_) were determined at α1β3γ2 type (Figure 5). On α1β2γ2 and α3β2γ2 receptors at concentration of 10 μM, Tx-NM3-1 almost completely inhibited GABA-evoked currents (Figure 4b). Two other toxins Tx-NM4 and Tx-NM2 were less effective and Tx-NM3-2 showed extremely low activity (Figure 4b). Interestingly, the efficiency of interactions of Tx-NM3-1, Tx-NM3-2, and Tx-NM4 with two GABA_A_R subtypes (α1β2γ2 and α3β2γ2) was different and the largest difference was observed for Tx-NM4 (Figure 4b).

More detailed studies of activity were performed at α1β3γ2 GABA_A_R. The dependences of GABA-elicited currents on toxin concentrations were investigated and IC_50_ values were determined. The inhibition curves for the most active toxins Tx-NM2 and Tx-NM3-1 are shown in Figure 5.

The data for all four toxins studied are given in the Table 2. On this receptor subtype, Tx-NM3-1 was the most active, followed by Tx-NM2. Tx-NM4 was one order of magnitude less active than the last two toxins, and Tx-NM3-2 manifested only very weak activity.

Summing up all the data concerning the action of the *N. melanoleuca* TFTs on GABA_A_Rs, we can conclude that Tx-NM3-1 was the most active, Tx-NM2 was a little bit less active, Tx-NM4 manifested very weak activity, and Tx-NM3-2 was practically inactive.

#### 2.2.2. Interaction with nAChRs

**Figure 6:**
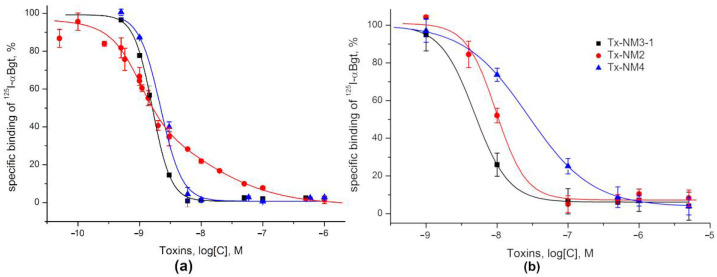
Competition of *N. melanoleuca* toxins with ^125^I-*α*Bgt for binding to *Torpedo* (**a**) and human *α*7 nAChR expressed in the GH_4_C_1_ cell line (**b**). Each data point is presented as the mean of three independent experiments ± S.E.

The binding of isolated toxins to nAChRs was studied by competitive radioligand method using radioactive α-Bgt (^125^I-αBgt) as a ligand. Interaction with nAChRs of muscle type from *Torpedo* electric organ and of human α7 type heterologously expressed in GH_4_C_1_ cells was investigated. It was found that Tx-NM2, Tx-NM3-1, and Tx-NM4 efficiently inhibited binding to both receptor subtypes (Figure 6). IC_50_ values for studied toxins were in the nanomolar range (Table 3).

Interestingly, at *Torpedo* nAChR, the experimental data for Tx-NM2 differed greatly from those for Tx-NM3-1 and Tx-NM4 and fit two-site binding model with fairly smooth inhibition curve (Figure 6a). The difference in affinity of Tx-NM2 to these binding sites was about one order of magnitude (1 nM versus 8.66 nM) (Table 3). The affinities of Tx-NM3-1 and Tx-NM4 to *Torpedo* nAChR were almost similar, Tx-NM3-1 being a slightly more active. At the α7 nAChR, the most active was Tx-NM3-1 with IC_50_ value of 4.84 nM, while Tx-Nm2 and Tx-NM4 were less active manifesting IC_50_ values of 13.02 and 26.89 nM, respectively (Figure 6b). At a concentration of 1 µM, toxin Tx-NM3-2 inhibited ^125^I-*α*Bgt binding by only about 40% and was not analyzed further.

#### 2.2.3. Interaction with Acetylcholine Binding Proteins from *Lymnaea stagnalis* and *Aplysia californica*

**Figure 7:**
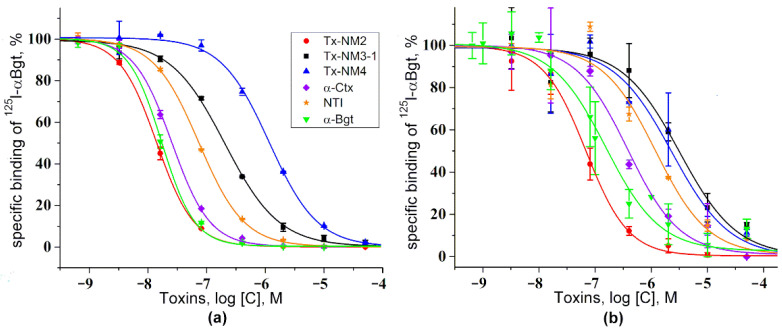
Competition of TFT neurotoxins with ^125^I-*α*Bgt for binding to acetylcholine binding proteins (AChBPs) from *L. stagnalis* (**a**) and *A. californica* (**b**). The results of three (on *L. stagnalis* AChBP) or four (on *A. californica* AChBP) independent experiments are shown. The exception is Tx-MN4, for which two independent experiments on *L. stagnalis* AChBP were carried out due to insufficient amount of material for the three experiments. Each data point is presented as the mean ± S.E. NTI—long type neurotoxin I from cobra *N. oxiana*.

Competitive radioligand assay with ^125^I-αBgt was applied also for testing the interaction of *N. melanoleuca* toxins with acetylcholine binding proteins (AChBPs) from *L. stagnalis* and *A. californica*. The results obtained showed that Tx-NM2 was the most active (Figure 7). The affinity of this toxin to both proteins exceeded that of α-Bgt and α-Ctx (Table 4). However, the affinity of Tx-NM3-1 and Tx-NM4 to both proteins was the lowest among the toxins studied (Figure 7, Table 4).

### 2.3. Molecular Modelling

**Figure 8:**
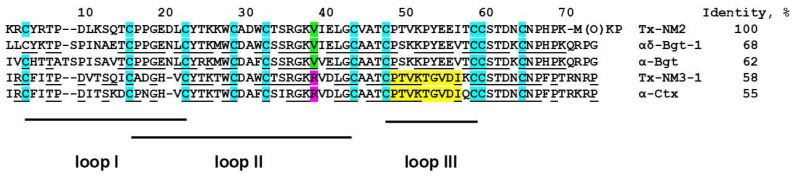
Comparison of Tx-NM2 amino acid sequence with those of other long chain neurotoxins. αδ-Bgt-1 is αδ-bungarotoxin-1 from *B. candidus* venom (accession number A1IVR8 in UniProtKB). The amino acid residues identical to those in Tx-NM2 are underlined. The loop III fragments identical in Tx-NM3-1 and α-Ctx are marked in yellow.

The efficiency of Tx-NM3-1 and Tx-NM4 interactions with α1β3γ2 GABA_A_R differs more than an order of magnitude (Table 2). However, the amino acid sequences of these toxins differ only in five positions—three located at the tips of loops II and two at the tip of loop III. This may suggest the involvement of the loop III in interaction with GABA_A_R, in addition to loop II the importance of which for binding was shown earlier [15]. Interestingly, the amino acid sequences of Tx-NM3-1 and α-Ctx are practically identical in this loop III region: positions 49–58 in Figure 8. To find structural elements which may explain the differences in the interaction of *N. melanoleuca* toxins with GABA_A_R, we performed the molecular modeling of toxin spatial structures and structure of the complex between GABA_A_R and toxin.

**Figure 9:**
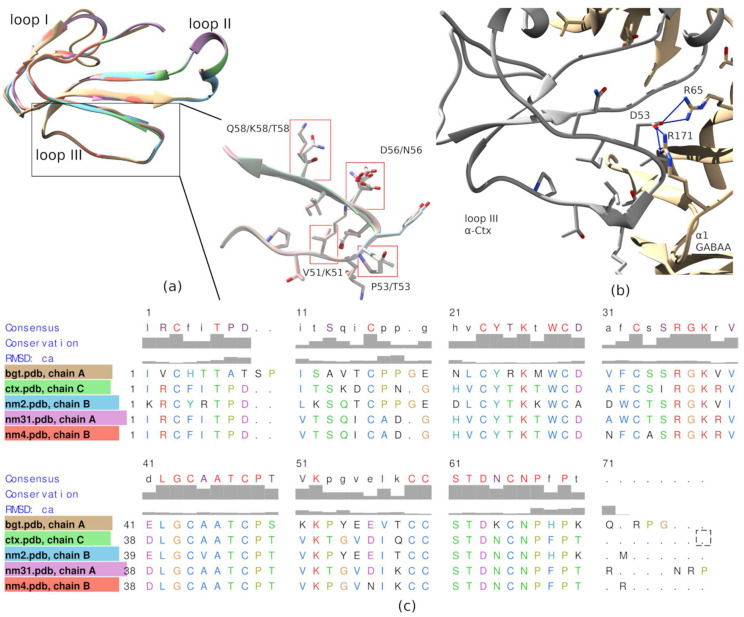
Homology modeling of *N. melanoleuca* neurotoxins. (**a**) Superposition of polypeptide chains of neurotoxins and a zoomed view of the molecular models of loop III of α-Ctx, α-Bgt, and *N. melanoleuca* neurotoxins. Amino acid residues differing in these TFTs are highlighted. Color code of the respective toxins is shown on the panel (**c**) in the headers of the respective sequences. (**b**) A view of the molecular model of complex formed by loop III of α-Ctx with the orthosteric site of the GABA_A_R. Asp56 from the α-Ctx loop III forms hydrogen bonds (shown as light blue sticks) with Arg65 and Arg171 of the α1 subunit. (**c**) Sequence alignment of *N. melanoleuca* neurotoxins with α-Ctx and α-Btx, based on alignments of spatial structures in UCSF Chimera “match-align” tool.

The models of *N. melanoleuca* toxin structures were constructed by homology modeling in Swissmodel service using α-Ctx structure (PDB 1YI5) as a template. The loop III structures for *N. melanoleuca* toxins are quite similar (Figure 9a).

**Figure 10:**
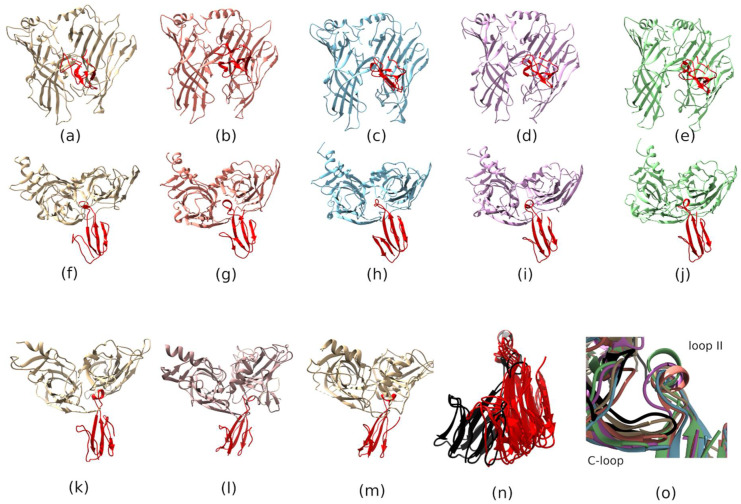
Molecular modeling of TFTs in complexes with GABA_A_R. (**a**–**e**) Side views of different TFT complexes with GABA_A_R extracellular domains; (**f**–**j**) views of different TFT complexes with GABA_A_R from the extracellular space; (**a**,**f**) show α-Bgt complexes; (**b**,**g**) α-Ctx; (**c**,**h**) Tx-NM2; (**d**,**i**) Tx-NM3-1; (**e**,**j**) Tx-NM4. (**k**–**m**) Views of TFT complexes with nAChRs and AChBP from the extracellular space: (**k**) α-Bgt in complex with muscle nAChR (PDB 6UWZ); (**l**) α-Bgt in complex with α7 nAChR extracellular domain (PDB 4HQP); (**m**) α-Ctx in complex with AChBP (PBD 1YI5). (**n**) Superposition of TFT structures in complexes with nAChRs (black) and in modeled complexes with GABA_A_R (red). Toxins in complexes with GABA_A_R show tilt toward complementary subunit. (**o**) Comparison of loop II positions in different models (color code is the same as on the Figure 9). Position of loop C in the cryo-EM structure of the GABA_A_R with the non-peptide antagonist bicuculline (PDB 6HUK) is shown in black.

Resulting homology models were submitted to protein docking Tox Dock instrument on the Rosetta server [26,27] to generate plausible starting structures of the toxins-GABA_A_R complexes. Flexibility of toxin molecules and receptors were taken into account and model structures were subsequently subjected to short molecular dynamics using GROMACS 5.0 package [28]. The resulting structures were visualized and inspected in UCSF Chimera [29] (Figure 10). All models showed similar positions with the TFT’s loop II buried under the C-loop of beta3-subunit of GABA_A_R (Figure 10a–e for the side view and Figure 10f–j for the top view from the extracellular side). Modeled complexes were compared to published X-ray structures of α-Bgt in complex with α7 nAChR/AChBP chimera [30] (Figure 10l) and of α-Ctx in complex with AChBP [31] (Figure 10m) as well as cryo-EM structures of α-Bgt in complex with muscle nAChR [32] (Figure 10k). Structures of principal subunits (α1 and α7 nAChRs, AChBP(+) and β3 GABA_A_R) of all mentioned above complexes were aligned in UCSF Chimera and TFT positions relative to the receptor were inspected. Interestingly, all putative complexes with GABA_A_R showed TFT position tilted towards the complementary subunit as compared to nAChR complexes (Figure 10n). In such tilted position the loop III of TFT molecule can form contacts with the complementary subunit in the GABA_A_R binding site.

The positions toxin loops II placed to the orthosteric GABA_A_R ligand binding site show a remarkable correlation with the functional inhibitory activity of the modeled toxins. As follows from our measurements, all tested TFTs can be ranked according to their inhibitory activity toward GABA_A_R in the following way: α-Ctx = Tx-NM3-1 > Tx-NM2 > Tx-NM4 > α-Bgt. On the other hand, distances between β-carbons of Thr 202 located at the tip of loop C of β3 subunit and Arg 65 located on the complementary side of the orthosteric ligand binding site of the receptor α1 subunit are as follows: for α-Ctx—17 Å, Tx-NM3-1—15 Å, Tx-NM2—14 Å, Tx-NM4—13 Å, and α-Bgt—12 Å. The distance between these atoms may be used as a simple metric of loop C closure upon ligand binding and may be a possible predictor of the inhibitory activity of the TFT. The stronger toxin is bound to receptor and the deeper inserted in the binding pocket, the longer is distance between the indicated amino acid residues. However, this may not be true for low molecular ligands. Thus, cryo-EM structure of GABA_A_R with non-peptidic antagonist bicuculline (PDB 6HUK) is characterized by the even shorter distance between β-carbons of the above mentioned Thr and Arg residues, that is only 10 Å.

According to molecular modeling of the α-Ctx-GABA_A_R complex performed earlier [15], loop III contacts GABA_A_R through Asp56 salt bridges with Arg65 and Arg171 of the α1 subunit and Thr53 forming hydrogen bond with Val177 backbone of the α1 subunit. Asp56 is present in the amino acid sequence of Tx-NM3-1 but is replaced by glutamic acid in α-Bgt and Tx-NM2 and by asparagine in Tx-NM4 (Figure 9b,c). Thr53 present in α-Ctx and Tx-NM3-1 is replaced by proline in α-Bgt, Tx-Nm2, and Tx-Nm4. These replacements might substantially weaken the interaction of toxins with GABA_A_R. However, further more extensive molecular modeling and structure–function relationship studies are needed to investigate details of TFT-GABA_A_R pharmacophore in details.

## 3. Discussion

Earlier it was shown that several snake venom toxins blocked GABA_A_R [13,14,15]. We have found that the snake venom toxins manifested a different efficiency of the interaction with the receptor, α-Ctx being the most active [15]. As mentioned in the introduction, we suggested that the Arg36 present in α-Ctx (Arg39 in the alignment given in Figure 2) might determine the highest activity of this toxin against GABA_A_R. To check if this is true, we decided to compare the activity of toxins which differ at this position and for this purpose isolated toxins from *N. melanoleuca* cobra venom. Two long type α-neurotoxins were isolated earlier from this venom: long neurotoxin 1 (P01383) [24] containing valine residue at the position 38 (Figure 2), and long neurotoxin 2 (P01388) [23] with Arg38 in the sequence (Figure 2). The only information about biological activity available for these toxins was LD_50_ of 1.5 mg/kg for neurotoxin 1 by intraperitoneal injection [24]. We wanted to get these particular toxins, but in our work we isolated 4 toxins with molecular masses in the range of 7–8 kDa, characteristic for long type α-neurotoxins (Table 1). Their analysis by high resolution mass spectrometry showed that one of the isolated toxins, designated by us as Tx-NM4, was identical to long neurotoxin 2, while toxin Tx-NM3-1 was long neurotoxin 2 analogue with substitutions in five positions (Figure 2). Tx-NM2 represented an analogue of *N. melanoleuca* long neurotoxin 1 (P01383) and Tx-NM3-2 was homologous to muscarinic toxin-like protein 1 (MTLP-1, P82462) from *N. kaouthia* cobra venom. The differences between the published amino acid sequences and those determined in the present work may be explained by different geographical origin of snakes from which the venoms were obtained.

The biological activity of the isolated toxins was assessed against several molecular targets. In the studies on GABA_A_R, competition experiments with fluorescently labeled α-Ctx and electrophysiological assays were carried out using several combinations of receptor subunits. Both methods gave the consistent results. Toxin Tx-NM3-1 was the most active on all GABA_A_R subtypes studied and manifested activity similar to that of α-Ctx. However, at α1β3γ2 receptor subtype Tx-NM3-1 was less active than α-Ctx displaying IC_50_ of 680 nM in contrast to 236 nM for α-Ctx [15]. Tx-NM2 was slightly less active than Tx-NM3-1 showing IC_50_ of 1.25 µM at α1β3γ2 GABA_A_R. Tx-NM4 was a weaker antagonist than Tx-NM2 and Tx-NM3-1 at all subunit combinations, while Tx-NM3-2 was practically inactive. At the inhibition of α1β3γ2 GABA_A_R, the difference in affinity between the most active Tx-NM3-1 and the least active Tx-NM4 was more than one order of magnitude. Tx-NM4 at 10 µM more potently inhibited α1β2γ2 than α3β2γ2 GABA_A_R. Thus, the earlier suggestion about essential role of Arg36 (Arg39 in Figure 2 and Figure 8) in α-Ctx binding to GABA_A_R was not confirmed, as both Tx-NM3-1 and Tx-NM4 contain arginine residue at this position. The analysis of toxin amino acid sequences revealed the identity of loop III sequence in α-Ctx and Tx-NM3-1. Molecular modeling of α-Ctx-GABA_A_R complex revealed the contacts between loop III of α-Ctx and α1 subunit of GABA_A_R. It was found that Asp56 of α-Ctx loop III forms salt bridges with Arg65 and Arg171 of the α1 subunit, and Thr53 forms a hydrogen bond with Val177 backbone of the α1 subunit (Figure 9b). The aspartic acid and threonine residues are present at these positions in Tx-NM3-1 as well. The interaction of these residues with α1 subunit of GABA_A_R may contribute to the stronger binding of α-Ctx and Tx-NM3-1 to the receptor.

Tx-NM3-2 is the muscarinic toxin-like protein and its amino acid sequence differs greatly from those of the long type neurotoxins to which other studied toxins belong. The muscarinic toxin-like protein was tested on GABA_A_R for the first time and it showed some activity, albeit very weak, against α3β2γ2 GABA_A_R (Figure 4). This is the first indication of the activity of muscarinic toxin-like protein against GABA_A_R.

The activity of all isolated toxins was studied in competition experiments for binding to *Torpedo* and α7 nAChRs as well as to two AChBPs. The neurotoxins tested showed a relatively high affinity to both receptor subtypes. It was found that at *Torpedo* nAChR, Tx-NM2 distinguished two binding sites, the affinity differing by about an order of magnitude (Table 3). Earlier, it was shown that the two binding sites in *Torpedo* and muscle types nAChRs were distinguished by αδ-bungarotoxins from the krait *B. candidus* venom [12], the difference being 17-fold for αδ-bungarotoxin-1. Comparison of the amino acid sequences of *N. melanoleuca* toxins with those of bungarotoxins and α-Ctx showed that the sequence of Tx-NM2 has a higher identity to that of αδ-bungarotoxin-1 than to α-Btx and α-Ctx (69% versus 61 and 55%, respectively, Figure 8). At the same time Tx-NM3-1 has 81% identical residues with α-Ctx and only 50% with αδ-bungarotoxin-1. Moreover, the sequence characteristics that were supposed to be responsible for difference in activity between α-Btx and αδ-bungarotoxin-1, i.e., the shortened loop I, the change of Phe residue in position 32 to Trp as well as Arg25 to Thr, are present in Tx-NM2. In addition, only Tx-NM2 and αδ-bungarotoxin-1 contain Tyr residue in position 4 and a positively charged residue in position 5. This similarity in structural features and activity between Tx-NM2 and αδ-bungarotoxin-1 supports the earlier ideas [12] on the capacity of long chain neurotoxin to distinguish two binding sites in the muscle-type nAChRs. It was found that Tx-NM3-1 was the most active against α7 nAChR (IC_50_ 4.84 nM), followed by Tx-NM2 and Tx-NM4 with IC_50_ of 13.02 and 26.89 nM, respectively. Although the affinities of these toxins for nAChR are much higher than for GABA_A_R, their rank coincides with that observed for GABA_A_R suggesting that both receptors might have similar structural elements involved in toxin binding to them.

AChBPs are considered as models of the extracellular domains of Cys-loop receptors, in particular of nAChRs. Interestingly, AChBPs has only 20–24% sequence identity with nAChRs, however their pharmacological properties are similar to those of nAChRs. Studies of the binding of *N. melanoleuca* toxins to AChBPs from *L. stagnalis* and *A. californica* showed that toxin Tx-NM2 possessed the highest affinity to both proteins, while toxins Tx-NM3-1 and Tx-NM4 were several orders of magnitude less active. On *A. californica* AChBP, Tx-NM3-1 was the least active, which is very different from its activity against nAChR and GABA_A_R, where it was most active. This once again highlights the differences between AChBPs and the extracellular domains of nAChR and the GABA_A_R.

Considering the above data for nAChRs and AChBPs, one can conclude that for these targets, *N. melanoleuca* toxins manifest affinities similar to those of α-Btx and α-Ctx, and Tx-NM2 on AChBP from *L. stagnalis* showed the affinity higher than these two neurotoxins.

To obtain some information about possible molecular mechanisms determining the specificity of TFT interaction with GABA_A_R, we performed molecular modeling of the complexes formed by *N. melanoleuca* toxins, α-Bgt and α-Ctx with GABA_A_R. The modeling showed that the orientation of toxin molecules at GABA_A_R and nAChRs were different and toxin molecules in complexes with GABA_A_R tilted towards the complementary subunit. This tilt can lead to close contact of toxin loop III with the α1 subunit of the receptor. More detailed consideration revealed the amino acid residues which can form salt bridges (Asp56 in α-Ctx) and hydrogen bond (Thr53 in α-Ctx) with α1 subunit. These amino acid residues are present in the amino acid sequence of Tx-NM3-1, interacting with GABA_A_R with affinity similar to that of α-Ctx, but are replaced by other residues in less active toxins. Such substitutions may lead to a significant decrease in the efficiency of toxin interactions with GABA_A_R.

## 4. Conclusions

In our previous paper [15] it was found that among several snake toxins studied only α-Ctx manifested high activity against GABA_A_R and we put forward the hypothesis about essential role of Arg36 as the determinant of high affinity to GABA_A_R. To check this hypothesis, in this work we additionally studied several snake toxins for their ability to interact with GABA_A_R. For this purpose, four toxins were isolated from African cobra *N. melanoleuca* venom and their amino acid sequences were established by mass spectrometry. The amino acid sequence of one toxin was identical to that of previously known *N. melanoleuca* long neurotoxin 2. The second toxin sequenced differed from that of neurotoxin 2 in five positions. The third one was homologous to *N. melanoleuca* long neurotoxin 1; its sequence differed from that of neurotoxin 1 in three positions. One more toxin was homologous to muscarinic toxin-like protein from *N. kaouthia* venom and this is the first muscarinic toxin-like protein isolated from African cobra venom. The interaction of *N. melanoleuca* toxins with *Torpedo* and α7 nAChRs as well as with AChBPs and several subtypes of GABA_A_Rs was studied. One of isolated toxins, being the most active on AChBPs, manifested different affinity to two binding sites on *Torpedo* nAChR. Together with earlier data [12] this may indicate that there is a group of long type α-neurotoxins capable to bind with different affinity to two binding sites in the muscle type nAChR. All *N. melanoleuca* toxins interacted with the GABA_A_R much weaker than with the nAChR: one neurotoxin was almost as active as α-Ctx, while others manifested lower activity. The earlier hypothesis about essential role of Arg36 as the sole determinant of high toxin affinity to GABA_A_R was not confirmed, but the results of molecular modeling suggest that the loop III may contribute to the efficient interaction of some long-chain neurotoxins with GABA_A_R. Experimental proof of the modeling data will be the task of our future work.

## 5. Materials and Methods

### 5.1. Materials

All salts obtained from local suppliers were of analytical grade or higher. Venom of cobra *N. melanoleuca* was from Latoxan (Valence, France). Acetonitrile was purchased from Catrosa Reaktiv LLC (Moscow, Russia), and trifluoroacetic acid from Merck KGaA (Darmstadt, Germany). GH_4_C_1_ cells transfected with hα7 nAChR cDNA were a gift of the Eli-Lilly Co. (London, UK). Muscle-type nAChR-enriched membranes from the electric organs of *Torpedo californica* were kindly provided by Prof. F. Hucho (Free University of Berlin, Germany). Acetylcholine binding proteins from *L. stagnalis* and *A. californica* were from Prof. A.B. Smit (Faculty of Earth and Life Sciences, Vrije Universiteit, Amsterdam).

### 5.2. Isolation of Neurotoxins

A 600 mg sample of dried *N. melanoleuca* venom was dissolved in 0.1 M ammonium acetate buffer, pH 6.2, and applied to a Sephadex G50s column (4.5 × 150 cm) equilibrated in the same buffer. The column was eluted at flow rate 32 mL/min. The fractions obtained were pooled as shown in Figure 1a. Fraction 6 was further separated on a HEMA BIO 1000CM column (4 × 250 mm) (Tessek, Prague, Czech Republic) in an ammonium acetate gradient from 5 to 500 mM (pH 7.5) in 100 min at flow rate 1.0 mL/min (Figure 1b). Fractions 2, 3 and 4 were freeze-dried and further purified by reversed phase chromatography on Jupiter C18 column (10 × 250 mm, Phenomenex, Torrance, CA, USA) in in a gradient of acetonitrile 20–35% in 60 min in the presence of 0.1% trifluoroacetic acid, at a flow rate of 2.0 mL/min (Figure S1). After freeze-drying, the obtained proteins were used for further studies.

### 5.3. Mass Spectrometry Analysis

For mass spectrometry measurements, the carbamidomethylated toxins were digested with trypsin and chymotrypsin at a 1:50 (*w*/*w*) ratio overnight at 37 °C. Desalting of peptides was carried out using SDB-RPS StageTips that were prepared as described earlier [33]. After overnight digestion, peptide solution was acidified by equal volume of 2% (*v*/*v*) TFA and peptides were loaded on SDB-RPS StageTip by centrifugation at 200× g. StageTip was washed by 50 µL 0.2% (*v*/*v*) TFA and peptides were eluted by 50 μL 50% (*v*/*v*) acetonitrile, 5% (*v*/*v*) ammonia, lyophilized, and stored at −80 °C. Before analyses, peptides were dissolved in 20 µL of 2% (*v*/*v*) acetonitrile, 0.1% (*v*/*v*) TFA, and sonicated for 2 min. Samples were loaded to a home-made trap column 20 × 0.1 mm, packed with Inertsil ODS3 3 μm sorbent (GLSciences, Tokyo, Japan ), in the loading buffer (2% ACN, 98% H_2_O, 0.1% TFA) at 10 μL/min flow and separated at RT in a home-packed [34] fused-silica column 300 × 0.1 mm packed with Reprosil PUR C18AQ 1.9 (Dr. Maisch, Ammerbuch-Entringen, Germany) into an emitter prepared with P2000 Laser Puller (Sutter Instrument, Novato, CA, USA). Reverse-phase chromatography was performed with an Ultimate 3000 Nano LC System (Thermo Fisher Scientific, Waltham, MA, USA), which was coupled to a Q Exactive Plus benchtop Orbitrap mass spectrometer (Thermo Fisher Scientific) via a nanoelectrospray source (Thermo Fisher Scientific). Peptide samples were eluted with a linear gradient of 80% ACN, 19.9% H_2_O, 0.1% FA (buffer B) in 99.9% H_2_O, 0.1% FA (solvent A) from 4 to 36% of solvent B in 60 min at 0.5 μL/min flow, intact toxins were separated by linear gradient from 10 to 60% of solvent B in 18 min at 0.5 μL/min flow.

MS raw files were analyzed by PEAKS Studio 8.5 (Waterloo, ON, Canada) [35] and peak lists were searched against Serpentes Uniprot-Tremble FASTA (canonical and isoform) database version of May 2018 (144954 entries) with cysteine carbamidomethylation as a fixed modification and methionine oxidation and asparagine and glutamine deamidation as variable modifications. Enzyme specificity in the database search was set to trypsin with semi-specific digest mode. False discovery rate was set to 0.01 for peptide-spectrum matches and was determined by searching a reverse database. Peptide identification was performed with an allowed initial precursor mass deviation up to 10 p.p.m. and an allowed fragment mass deviation 0.05 Da.

### 5.4. Expression of GABAAR in Xenopus Oocytes

*Xenopus laevis* oocytes were prepared as described [36]. The work with oocytes was approved by the Shemyakin-Ovchinnikov Institute of Bioorganic Chemistry RAS with the approval number IACUC 251/2018 26.02.18. Plasmid DNAs encoding rat GABA_A_ receptor subunits in pCI mammalian expression vector (Promega, Madison, WI, USA) were kindly provided by Dr. M. Ernst from the Medical University of Vienna. Next day after harvesting oocytes were injected by means of Nanoject II (Drummond Scientific, Broomall, PA, USA) with 2 ng mixture containing vector DNAs encoding receptor α1 or 3, β2 or 3, and γ2 subunits at 1:1:10 mass ratio. Injected oocytes were incubated at 18 °C for 2–3 days in ND96 solution (5 mM HEPES/NaOH pH 7.4, 96 mM NaCl, 2 mM KCl, 1.8 mM CaCl_2_, 2 mM MgCl_2_) supplemented with gentamycin at 40 μg/mL.

### 5.5. Two-Electrode Voltage Clamp Electrophysiology Assay

Oocyte was placed in the flow through chamber combined with a nylon grid holding the bath of ND96 solution. Membrane potential of oocyte was clamped at −60mV by TURBO TEC-03X (npi electronic GmbH, Tamm, Germany). Electrodes were pulled from borosilicate capillary (Warner Instruments, Holliston, MA, USA) and filled with 3M KCl solution. Currents were recorded and digitized by WinWCP (University of Strathclyde, Glasgow, UK) software. After stable amplitude of 10 μM GABA-evoked currents was obtained oocyte was pre-incubated with toxin ND96-based solution for three minutes followed by co-application with 10 μM GABA. After steady baseline potential was achieved usually in a five-minute washout session oocyte was perfused with GABA again for current amplitude stability control purposes and pre-incubated with next sample. All solutions were applied manually with automatic pipette in a volume of 200 μL (Eppendorf, Hamburg, Germany). To confirm γ subunit incorporation, GABA response in the presence of 50 μM Zn^2^⁺ and 1 μM diazepam was tested under the same conditions. Data for toxins are presented as the amplitude ratio of the currents elicited by 10 μM GABA in the presence of toxin to the average of the currents in control elicited by 10 μM GABA before co-application with toxin (100% control current amplitude) in the same oocyte. Data were collected from at least three oocytes from three different batches. Representative current traces, bar graphs and toxin-dependent dose response curves were plotted by Origin 8.1 (OriginLab, Northampton, MA, USA).

### 5.6. Mammalian Cell Culture

Mouse neuroblastoma Neuro2a cells (Russian collection of cell cultures, Institute of Cytology, Saint Petersburg, Russia) were routinely cultured in incubator (Sanyo, Osaka, Japan) at 37 °C and 5% CO_2_ in tissue culture treated T25 flasks (SPL, Pocheon, Korea) containing 5 mL Dulbecco’s modified Eagle’s medium (DMEM, Paneco, Moscow, Russia) supplemented with 10% fetal bovine serum (FBS, PAA Laboratories, Pasching, Austria), 50 units/mL streptomycin and 50 µg/mL penicillin. Cells were splitted 1:10 at confluency twice a week non-enzymatically using Versene solution (Paneco, Moscow, Russia).

### 5.7. Fluorescent Ligand Competition Assay

Neuro2a cells sub-cultured 1:5 24 h before transfection were growing on clear 96-well plate (Corning, Corning, NY, USA) in a complete DMEM (Paneco, Moscow, Russia). Lipofectamine (Invitrogen, Waltham, MA, USA) mediated transfection was performed with equal amounts (0.14 µg/mL) of pCI plasmid expression vectors encoding rat α1, β3, γ2 GABA_A_R subunits. Transfected Neuro2a cells were grown at 37 °C in 5% CO_2_ incubator for 72 h, at the day of experiment medium was substituted for extracellular solution containing (in mM) 140 NaCl, 2 CaCl_2_, 2.8 KCl, 4 MgCl_2_, 20 HEPES, 10 glucose at pH 7.4. Cells were pre-incubated with 10 µM of toxins for 15 min at room temperature followed by 20 min of incubation with 50 nM Alexa Fluor 546 α-Ctx conjugate in the final volume of 100 µL. Afterwards cells were washed 3 times with two-fold excess of extracellular solution. To control the level of non-specific fluorescence experiment with 10 µM α-Ctx was run under the same conditions. By means of epifluorescent microscope IX71 (Olympus, Tokyo, Japan) equipped with CCD camera pictures of 3 fields chosen on each plate well in bright-field illumination were taken. Fluorescence was counted using CellX and ImageJ open-source software. Intensity of the fluorescence was normalized on integral intensity of the plate well incubated in presence of 50 nM Alexa Fluor 546 α-Ctx conjugate. Each experimental point is an average of integral intensity independently measured on 6 plate wells from three separate passages ± SEM.

### 5.8. nAChR Competition Radioligand Assay

The competition binding assays with radio-iodinated α-bungarotoxin (^125^I-α-Bgt) were performed as in [37]. Briefly, suspension of GH_4_C_1_ cells stably transfected with human α7 nAChR (0.4 nM αBgt binding sites) were incubated in 50 μL binding buffer (20 mM Tris-HCl, pH 8.0, containing 1 mg/mL bovine serum albumin) for 90 min with various amounts of toxins. Thereafter, 0.1–0.2 nM ^125^I-α-Bgt (500 Ci/mmol) was added, and after an additional 5 min incubation, cell suspensions were applied to GF/C glass filters (Cytiva, Marlborough, MA, USA) pretreated with 0.3% polyethyleneimine. The samples were then washed (3 × 4 mL) with 20 mM cold Tris-HCl buffer, pH 8.0, containing 0.1 mg/mL bovine serum albumin and bound radioactivity was measured with a Wallac 1470 Wizard Gamma Counter (PerkinElmer, Waltham, MA, USA). Nonspecific ^125^I-*α*Bgt binding was determined in the presence of 200-fold excess of *α*-Ctx. The competition binding assays with AChBPs were performed as described in [38].

### 5.9. Molecular Modeling

The molecular models of *N. melanoleuca* toxin structures were constructed using free bioinformatic tool Swissmodel (https://swissmodel.expasy.org/ (accessed on 12 February 2021)). α-Ctx structure (PDB 1YI5) was used as template. The molecular model of α-Ctx-GABA_A_R complex was taken from [15]. Briefly, the model of orthosteric binding site was constructed basing on extracellular domain from X-ray structure of β3 GABA_A_R subunit [39]. Swissmoldel service was used to build the extracellular domain of α1 GABA_A_R subunit. RMSD between modelled α1 extracellular domain and recently published cryo-EM structure was calculated by UCSF Chimera Match maker tool and did not exceed 1.2 Å. Homology models were submitted to protein docking “Tox Dock” instrument on the Rosetta server [26,27] to generate structures of the toxin-GABA_A_R complexes. Flexibility of toxin molecules and receptors were taken into account and model structures were subsequently subjected to short molecular dynamics using GROMACS 5.0 package. Briefly, putative structures of TFT-GABA_A_R complexes obtained via “Tox Dock” were energy minimized using steepest descent minimization algorithm to a maximum force <1000 kJ/mol/nm. Short-range electrostatic and Van der Waals cut-offs were set to 1 Å, periodic boundary conditions were constructed with rhombododecaedron simulation box having 1.2 Å from protein image to the closest boundary. After energy minimization two consequent constrained 100 ps molecular dynamics were performed to equilibrate systems in NVT (constant number of particles, volume, and temperature) and NPT (constant number of particles, pressure, and temperature) conditions. Equilibration was followed by 100 ps of unconstrained molecular dynamics [28]. The resulting structures were visualized and inspected in UCSF Chimera [29]. To compare TFT positions “Match” and “Match-align” functions of UCSF Chimera were used.

## Figures and Tables

**Table 1:** Molecular masses of *N. melanoleuca* toxins.

Toxin	Determined Molecular Mass, Da	Calculated Molecular Mass, Da
Tx-NM2	8030.68	8030.69
Tx-NM3-1	7787.577	7787.602
Tx-NM3-2	7441.497	Not calculated
Tx-NM4	7756.581	7756.602

**Table 2:** IC_50_ values for inhibition of currents induced by GABA in α1β3γ2 GABA_A_R by *N. melanoleuca* toxins.

Toxin	IC_50_, µM
Tx-NM3-1	0.68 ± 0.14
Tx-NM2	1.25 ± 0.07
Tx-NM4	≈10
Tx-NM3-2	>>10

**Table 3:** IC_50_ values and Hill coefficients (n_H_) for inhibition of ^125^I-αBgt binding to *Torpedo* and human α7 nAChR by *N. melanoleuca* toxins.

Toxin	IC_50_, nM (CI 95% ^1^); n_H_ ± S.E.	
	*Torpedo* nAChR	α7 nAChR
Tx-NM2	1.01 (0.89–1.13); 1.92 ± 0.23 and 8.66 (8.31–9.04); 0.67 ± 0.05 ^2^	9.47 (8.56–10.49); 2.08 ± 0.31
Tx-NM3-1	1.61 (1.45–1.79); 2.88 ± 0.22	4.84 (4.36–5.36); 1.81 ± 0.12
Tx-NM4	2.17 (1.73–2.74); 2.5 ± 0.33	26.9 (25.84–27.98); 0.96 ± 0.01

^1^ CI 95%—95% confidence interval, ^2^ Two-site binding model.

**Table 4:** IC_50_ values for inhibition of ^125^I-αBgt binding to AChBPs by *N. melanoleuca* and other known TFT toxins.

Toxin	IC_50_, nM	
	AChBP *L. stagnalis*	AChBP *A. californica*
Tx-NM2	14.1 ± 0.1	68.6 ± 3.6
Tx-NM3-1	203 ± 10	3200 ± 600
Tx-NM4	1160 ± 40	2350 ± 450
α-Bgt	17.2 ± 0.7	155 ± 35
α-Ctx	25.4 ± 0.8	385 ± 45
NT-I ^1^	73.4 ± 1.3	1200 ± 300

^1^ Long type neurotoxin I from *N. oxiana.*

## Data Availability

All data is contained within this article and supplementary materials.

## Supplementary Materials

The following are available online at https://www.mdpi.com/2072-6651/13/2/164/s1, Figure S1: Separation of fractions 2 (**a**), 3 (**b**), and 4 (**c**) after ion exchange chromatography (Figure 1b) by reversed phase HPLC on Jupiter C18 column (10 × 250 mm, Phenomenex, Torrance, CA, USA) in a gradient of acetonitrile 20–35% in 60 min in the presence of 0.1% trifluoroacetic acid, at a flow rate of 2.0 mL/min. Figure S2: Extracted-ion chromatogram (XIC) from an LC-MS analysis and high-resolution spectra at z = 7 for toxins Tx-NM2, XIC at m/z = 1148.5–1149.5 (**a**); Tx-MN3-1, XIC at m/z = 1113.6–1114.6 (**b**); Tx-Nm3-2, XIC at m/z = 1064.3–1065.3 (**c**); and Tx-NM4, XIC at m/z = 1109.3–1110.3 (**d**).
