# Supplementary material for: Novel Three-Finger Neurotoxins from Naja melanoleuca Cobra Venom Interact with GABAA and Nicotinic Acetylcholine Receptors
Source: Toxins (Basel). 2021 Feb 20;13(2):164. doi: 10.3390/toxins13020164 (PMC7924340; doi:10.3390/toxins13020164)
Supplement: Supplementary file 1 [file toxins-13-00164-s001.pdf]

# Supplementary Materials: Novel Three-Finger Neurotoxins from *Naja melanoleuca* Cobra Venom Interact with GABAA and Nicotinic Acetylcholine Receptors

**Lina Son, Elena Kryukova, Rustam Ziganshin, Tatyana Andreeva, Denis Kudryavtsev, Igor Kasheverov, Victor Tsetlin and Yuri Utkin**

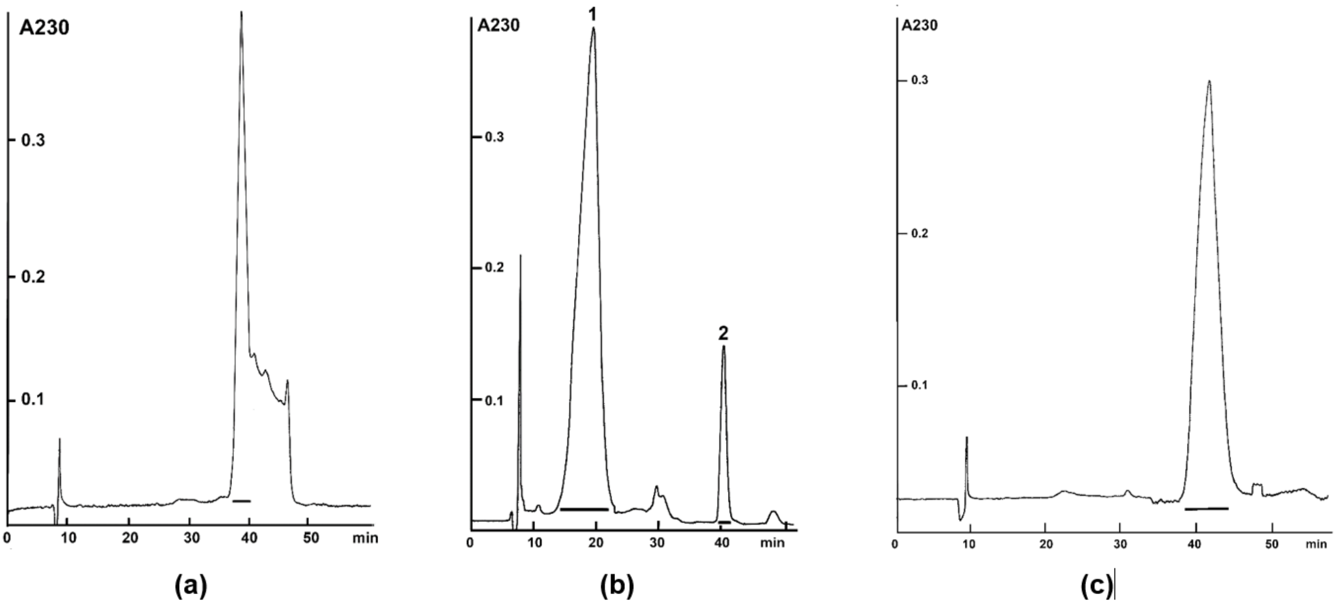


**Figure S1.** Separation of fractions 2 (**a**), 3 (**b**) and 4 (**c**) after ion exchange chromatography (Figure 1b) by reversed phase HPLC on Jupiter C18 column (10 × 250 mm, Phenomenex, Torrance, CA, USA) in a gradient of acetonitrile 20–35% in 60 min in the presence of 0.1% trifluoroacetic acid, at a flow rate of 2.0 mL/min. The horizontal bars indicate the fraction collected for Tx-NM2 (**a**), Tx-NM3-1 and Tx-NM3-2 (**b**), as well as Tx-NM4 (**c**).

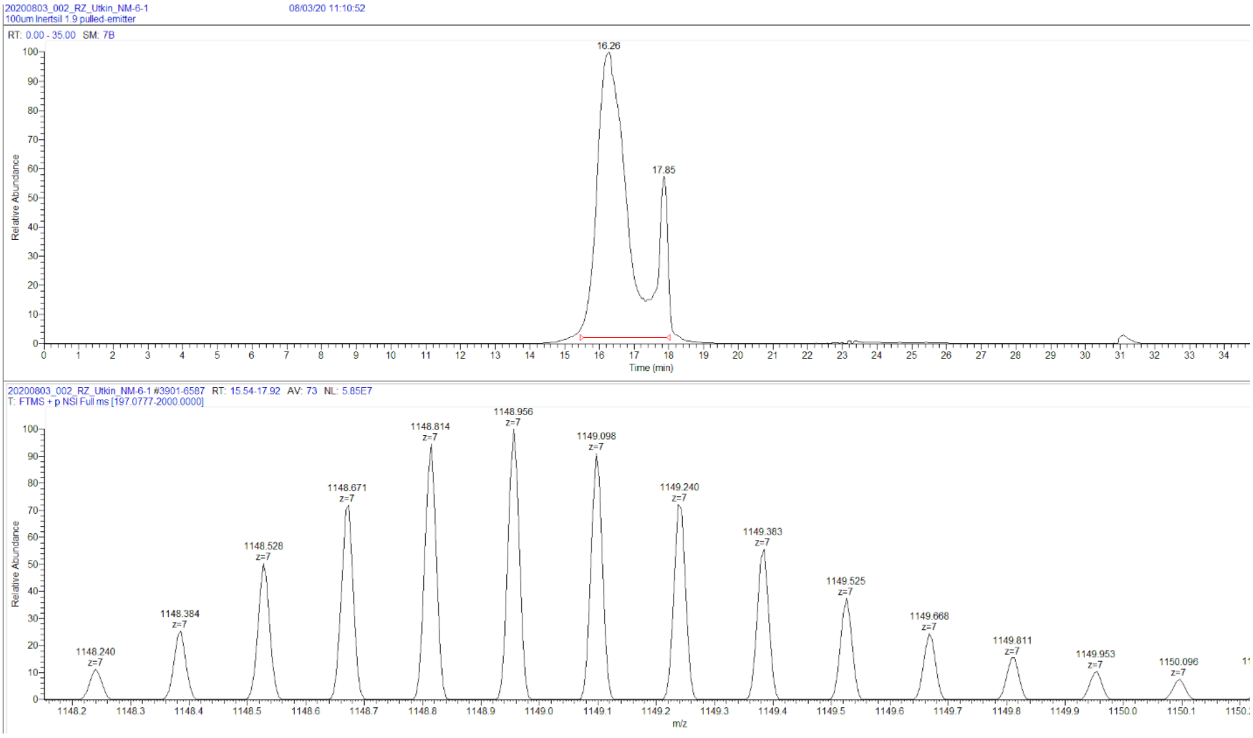
20200803_002_RZ_Utkin_NM-6-1
08/03/20 11:10:52
100um Inertsil 1.9 pulled-emitter
RT: 0.00 - 35.00 SM: 7B
16.26
17.85
Relative Abundance
Time (min)
20200803_002_RZ_Utkin_NM-6-1 #3901-6587 RT: 15.54-17.92 AV: 73 NL: 5.85E7
T: FTMS + p NSI Full ms [197.0777-2000.0000]
1148.240 z=7
1148.384 z=7
1148.528 z=7
1148.671 z=7
1148.814 z=7
1148.956 z=7
1149.098 z=7
1149.240 z=7
1149.383 z=7
1149.525 z=7
1149.668 z=7
1149.811 z=7
1149.953 z=7
1150.096 z=7
m/z

(a)

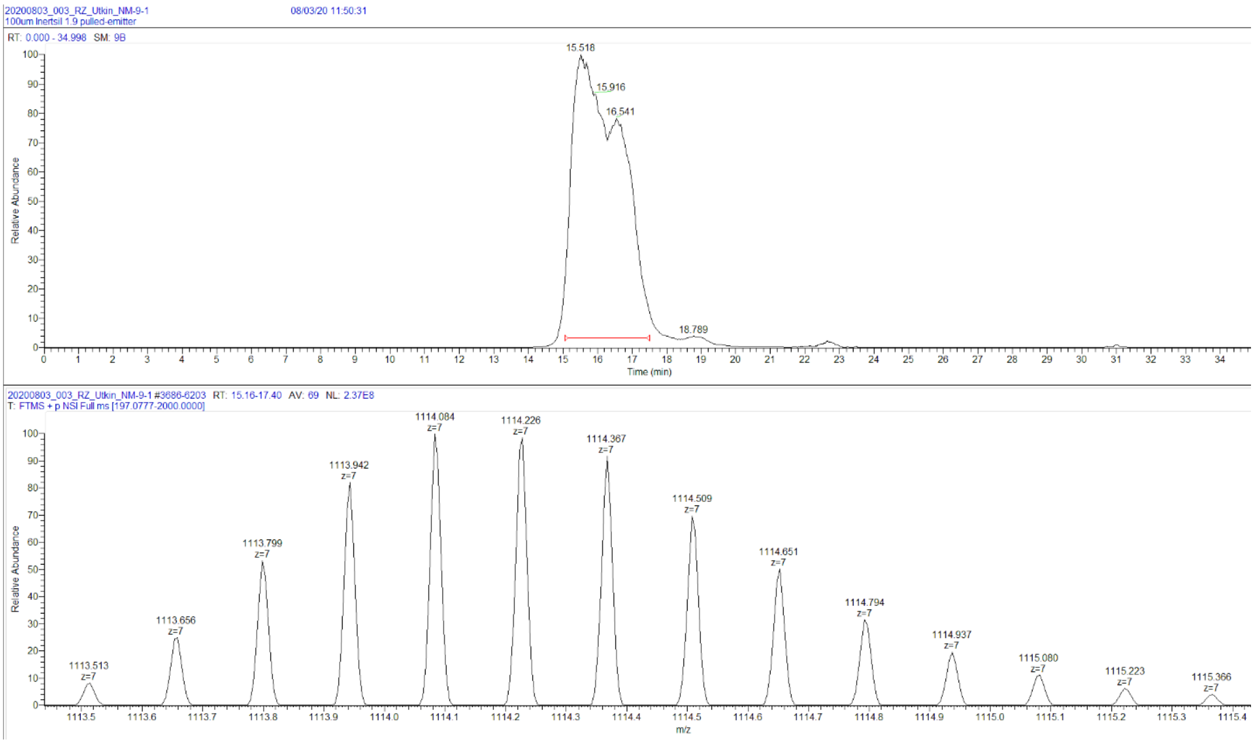
20200803_003_RZ_Utkin_NM-9-1
08/03/20 11:50:31
100um Inertsil 1.9 pulled-emitter
RT: 0.000 - 34.998 SM: 9B
15.518
15.916
16.541
18.789
Relative Abundance
Time (min)
20200803_003_RZ_Utkin_NM-9-1 #3686-6203 RT: 15.16-17.40 AV: 69 NL: 2.37E8
T: FTMS + p NSI Full ms [197.0777-2000.0000]
1113.513 z=7
1113.656 z=7
1113.799 z=7
1113.942 z=7
1114.084 z=7
1114.226 z=7
1114.367 z=7
1114.509 z=7
1114.651 z=7
1114.794 z=7
1114.937 z=7
1115.080 z=7
1115.223 z=7
1115.366 z=7
m/z

(b)

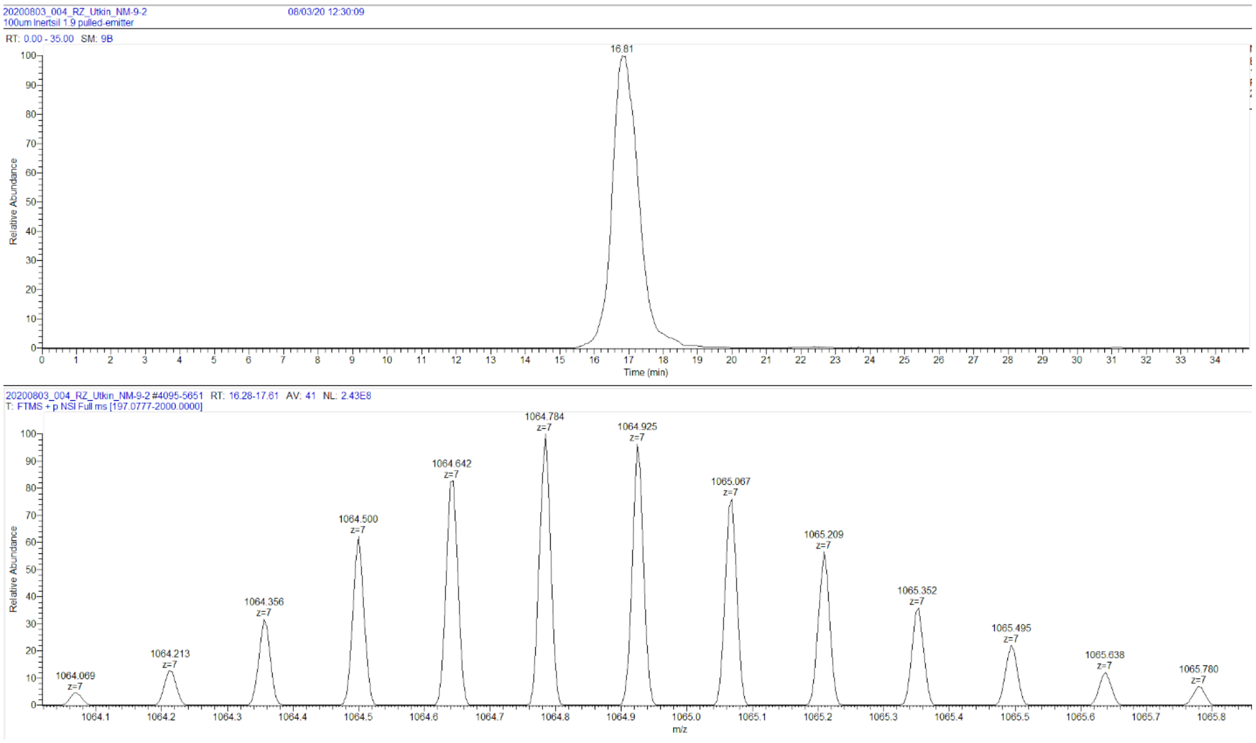


(c)

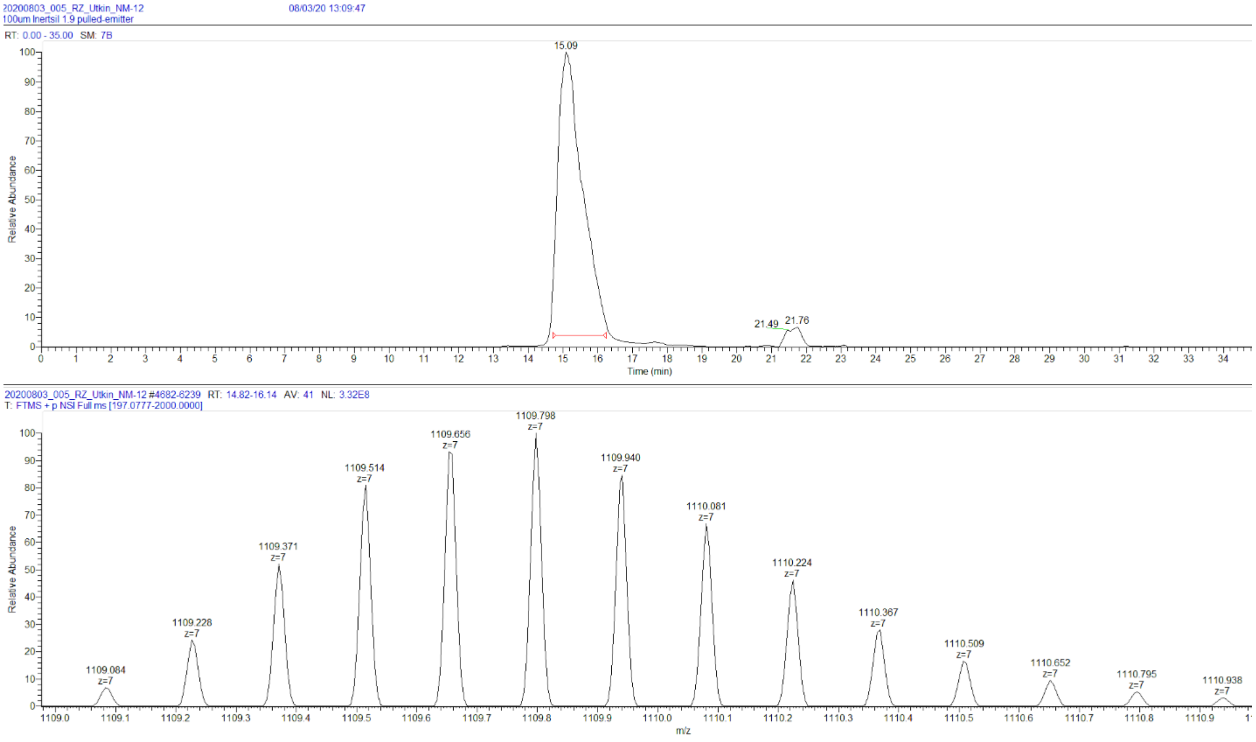


(d)

**Figure S2.** Extracted-ion chromatogram (XIC) from an LC-MS analysis and high-resolution spectra at z = 7 for toxins Tx-NM2, XIC at m/z = 1148.5–1149.5 (**a**); Tx-MN3-1, XIC at m/z = 1113.5–1114.5 (**b**); Tx-Nm3-2, XIC at m/z = 1064.5–1065.5 (**c**); and Tx-NM4, XIC at m/z = 1109.5–1110.5 (**d**). Each of two peaks observed for Tx-NM2 (**a**) and Tx-MN3-1 (**b**) give the same masses and represent toxin conformers. The signals with the lowest m/z ratio were used for the calculation of the monoisotopic mass by the following formula—protein mass = (m/z × z) – $M_H$ × z, where z is charge state equal to 7; $M_H$, atomic mass of proton equal to 0.998 a.u.
